# Asymmetric epileptic spasms after corpus callosotomy in children with West syndrome may be a good indicator for unilateral epileptic focus and subsequent resective surgery

**DOI:** 10.1002/epi4.12631

**Published:** 2022-08-01

**Authors:** Daiki Uchida, Tomonori Ono, Ryoko Honda, Yoshiaki Watanabe, Keisuke Toda, Shiro Baba, Takayuki Matsuo, Hiroshi Baba

**Affiliations:** ^1^ Department of Neurosurgery National Hospital Organization Nagasaki Medical Center Omura, Nagasaki Japan; ^2^ Department of Neurosurgery Nagasaki University Graduate School of Biomedical Sciences Nagasaki Japan; ^3^ Epilepsy Center National Hospital Organization Nagasaki Medical Center Omura, Nagasaki Japan; ^4^ Department of Pediatrics National Hospital Organization Nagasaki Medical Center Omura, Nagasaki Japan; ^5^ Department of Neurosurgery National Hospital Organization Nagasaki‐Kawatana Medical Center Kawatana, Nagasaki Japan; ^6^ Epilepsy Center Nishi‐Isahaya Hospital Isahaya, Nagasaki Japan

**Keywords:** corpus callosotomy, epilepsy surgery, epileptic spasms, west syndrome

## Abstract

**Objective:**

This retrospective study was designed to observe differences in ictal movements of epileptic spasm (ES) before and after corpus callosotomy (CC). We hypothesized that asymmetric expression of ES is more clarified after CC and would be a good indicator for the epileptic hemisphere.

**Methods:**

We selected 16 patients with intractable ES in West syndrome who were seizure‐free after CC and subsequent resection or disconnective surgery of the unilateral hemisphere. We retrospectively reviewed their behavioral ES recorded at video‐electroencephalography monitoring before and after CC. Asymmetric neck flexion (NF) and involuntary muscular contraction of the upper and lower extremities (MCU and MCL, respectively) were primarily described correlating their laterality and the responsible hemisphere proved by surgical resection.

**Results:**

Asymmetric NF, MCU, and MCL could be found both before and after CC. However, the percentage of those movements to the total number of ES increased after CC; asymmetric NF, 82.9% vs. 20.1%; unilaterally predominant MCU, 81% vs. 39.3%; and unilaterally predominant MCL, 77.6% vs. 29.9%. Regarding asymmetric NF, the direction in which the neck flexed or the head turned was significantly ipsilateral to the responsible hemisphere in 9 of 12 patients after CC (75%). The predominant side of MCU and MCL were significantly contralateral to the responsible hemisphere in 11 of 11 and 7 of 9 patients (100% and 77.8%, respectively).

**Significance:**

Asymmetric NF, MCU, and MCL were clarified in patients with ES who were successfully treated with CC and subsequent surgery. Those changes in ictal behaviors after CC may indicate the lateralization of epileptic activity and encourage more curative surgical treatment.

**Key Points**

- Video‐EEG data before and after corpus callosotomy (CC) were reviewed in patients whose epileptic spasm (ES) disappeared after subsequent resection or disconnective surgery.
- Symmetric ES before CC transformed into asymmetric patterns after CC.
- Direction of neck flexion was ipsilateral, and predominant muscular contraction of the upper and lower extremities was contralateral to the side of subsequent surgery.
- These asymmetric ES after CC was an important lateralizing sign indicating unilateral epileptic focus and subsequent surgery.

## 1 INTRODUCTION

West syndrome or infantile spasms is one of the most recognized epilepsy syndromes in early childhood.^1^ Epileptic spasm (ES) is the most disabling seizure in West syndrome, frequently pharmacoresistant and sometimes treated with surgery. Resection of the cortical lesion has been reported to lead to cessation of ES but that is primarily applied in a subset of patients with more discrete lesions exhibited on magnetic resonance imaging (MRI) and positron emission tomography (PET).^1^, ^2^, ^3^, ^4^, ^5^ Additionally, CC is an alternative option in patients with refractory generalized onset seizures including ES.^6^, ^7^, ^8^, ^9^, ^10^ The procedure is usually considered a palliative treatment for patients who are not candidates for resective surgery.^6^, ^11^ However, in recent years increased numbers of studies have documented that CC revealed an epileptic focus or hemisphere that had been unclear before surgery and brought a chance of seizure freedom by subsequent resective surgery in selected patients.^9^, ^10^, ^12^, ^13^, ^14^, ^15^ Therefore, finding candidates who can undergo such stepwise surgeries may be important.

Furthermore, ES is typically expressed as symmetric, but rarely asymmetric flexor, extensor, or mixed muscle contractions of the neck, trunk, and extremities, which are usually more sustained than a myoclonus seizure (up to 5 seconds) and more rapid than a tonic seizure.^1^, ^2^, ^16^, ^17^ However, it was reported that residual ES after CC is occasionally asymmetric in some patients (eg asymmetric muscular contraction of the neck and/or extremities).^12^ In this study, we retrospectively reviewed ES before and after CC, especially focusing on the laterality of neck flexion (NF) and involuntary muscular contraction of the upper and lower extremities (MCU and MCL, respectively) in patients whose ES disappeared after CC and subsequent resective surgery. We hypothesized that asymmetric expression of ES is clarified after CC and indicates lateralizing signs in patients who can undergo subsequent surgeries.

## 2 MATERIAL AND METHODS

This study was approved by National Nagasaki Medical Center Institutional Review Board (Approval number: 2021050).

### 2.1 Selection of patients

This study included 16 patients with medically refractory ES who were successfully treated with a combination of CC and subsequent resective surgery in the National Nagasaki Medical Center from 2006 to 2021. Resective surgeries also included multilobar disconnective surgeries and hemispherotomy.^12^ Clinical details are summarized in Table 1. Their age at epilepsy onset ranged from 2 to 22 months (median 5.5 months). Patients initially diagnosed with West syndrome and treated with appropriate medical treatments including adrenocorticotropic hormone and conventional antiseizure medicines, were referred to our center due to the inefficacy of those treatments. One patient had developed tonic seizures in addition to ES, and both seizures caused sudden head drop or fall (case 3). A preoperative comprehensive evaluation including video‐electroencephalography (EEG) monitoring, MRI, PET and/or single‐photon emission computed tomography (SPECT), and developmental and cognitive tests was done. Three patients had a specific MRI lesion (cases 2, 6, and 7), which comprised an indiscrete abnormality such as multilobar pachygyria with the blurring of the cortical‐subcortical boundary in two patients (cases 2 and 7) and a transmantle sign in the white matter in one (case 6). Additionally, seven patients had unilateral (cases 10–15) or bilateral (case 8) nonspecific lesions, including mild lobar or hemispheric volume loss and subtle signal changes in the white matter. EEG and PET/SPECT also showed laterality in some patients. Although these might be unilateral lesions suggesting a seizure focus, CC was firstly performed because a resectable epileptic focus was unidentified by ictal behavior and EEG.^10^, ^12^

**TABLE 1:** Patients' demographic data

Patient	Gender	Epilepsy syndrome	MRI	SPECT/PET before CC	EEG discharges before CC	SPECT/PET after CC	EEG discharges after CC	Subsequent RS	Age at CC/subsequent RS/(interval)
1	M	West syndrome	No lesion	Interictal SPECT Hypo (R‐F)	Bilateral, but predominantly L‐H	Ictal SPECT Hyper (R‐P)	R‐P	R parietal cortical resection	11 mo/25 m (14 m)
2	M	West syndrome	MCD (R‐P)	Interictal SPECT Hypo (R‐T)	Bilateral	Interictal SPECT Hypo (R‐H)	R‐H	R hemispherotomy	15 mo/30 mo (15 m)
3	F	West syndrome	No lesion	Interictal SPECT no laterality	Bilateral, but predominantly R‐H	PET Hypo (L‐H)	L‐H	L frontal cortical resection	50 mo/63 mo (23 m)
4	M	West syndrome	No lesion	Interictal SPECT no laterality	Bilateral	Ictal SPECT Hyper (R‐F) PET Hyper (R‐H)	R‐H, but predominantly R‐F	R frontal disconnection	15 mo/21 mo (6 m)
5	M	West syndrome	No lesion	Interictal SPECT no laterality	Bilateral	PET no laterality	R‐H	R subtotal hemispherotomy	40 mo/131 mo (91 m)
6	F	West syndrome	MCD (L‐P)	Interictal SPECT no laterality	Bilateral	PET Hypo (L‐H)	L‐H	L posterior quadrantectomy	49 mo/78 mo (29 m)
7	F	West syndrome	MCD (R‐P, ‐O)	Interictal SPECT no laterality	Bilateral	PET Hypo (B‐P, ‐O)	R‐P, ‐O	R posterior quadrantectomy	16 mo/45 mo (29 m)
8	M	West syndrome	Nonspecific (B‐F)	Interictal SPECT Hyper (R‐F)	Bilateral, but predominantly L‐H	PET Hypo (L‐H)	L‐H	L hemispherotomy	5 mo/10 mo (5 m)
9	M	West syndrome	No lesion	Interictal SPECT Hypo (R‐F)	Bilateral	PET Hypo (R‐H)	R‐H	R hemispherotomy	4 mo/16 mo (12 m)
10	M	West syndrome	Nonspecific (R‐F)	PET No laterality	Bilateral, but predominantly R‐H	PET Hypo (R‐H)	R‐F	R frontal disconnection	110 mo/121 mo (11 m)
11	F	West syndrome	Nonspecific (R‐F)	PET No laterality	Bilateral	PET Hypo (R‐H)	R‐F, ‐T, ‐P	R subtotal hemispherotomy	28 mo/45 mo (17 m)
12	M	West syndrome	Nonspecific (R‐F)	PET no laterality	Bilateral, but predominantly R‐H	PET Hypo (R‐H)	R‐F, ‐P	R hemispherotomy	5 mo/32 mo (27 m)
13	M	West syndrome	Nonspecific (L‐F)	PET Hypo (L‐H)	Bilateral, but predominantly L‐H	Ictal SPECT Hyper (L‐F) PET Hypo (L‐F, ‐P)	L‐F, ‐T	L frontal disconnection	36 mo/44 mo (8 m)
14	F	West syndrome	Nonspecific (R‐F)	PET Hypo (B‐T)	Bilateral, but predominantly L‐H	Ictal SPECT Hyper (R‐F, ‐P) PET Hypo (R‐H)	R‐F, ‐T, ‐P	R subtotal hemispherotomy	28 mo/41 mo (13 m)
15	F	West syndrome	Nonspecific (R‐H)	PET Hypo (R‐F, ‐T)	Bilateral, but predominantly L‐H	Interictal SPECT and PET Hypo (R‐H)	R‐F, ‐P	R frontotemporal disconnection	36 mo/51 mo (15 m)
16	M	West syndrome	No lesion	PET No laterality	Bilateral	PET Hypo (L‐H)	L‐H	L hemispherotomy	31 mo/45 mo (14 m)

Abbreviations: CC, corpus callosotomy; M, male; F, female; MRI, magnetic resonance imaging; MCD, malformation of cortical development; L, left; R, right; B, bilateral; F, frontal; T, temporal; P, parietal; O, occipital; H, hemispheric; Unspecific, unspecific findings including atrophy/volume loss of the brain region, signal changes in the white matter, and slight ventricular dilatation; SPECT, single‐photon emission computed tomography; PET, positron emission tomography; Hypo, hypoperfusion or hypometabolism; Hyper, hyperperfusion or hypermetabolism; EEG, electroencephalogram; RS, resective surgery including single‐ or multiple‐lobar disconnective surgery, posterior quadrantectomy, and hemispherotomy.

Furthermore, ES ceased or reduced in frequency immediately after CC but recurred during the follow‐up period. One patient with tonic seizures had only ES after CC (case 3). Therefore, medical treatments were modified in some patients, but ES could not be controlled. Long‐term video‐EEG monitoring was repeated as scheduled and showed asymmetric ES and lateralized epileptiform activities, suggesting explicitly localized or lateralized epileptic focus. Additionally, hypometabolism on PET and/or hyperperfusion on ictal SPECT coincided with the responsible region or hemisphere for those epileptic activities on EEG. Based on those findings and neurological conditions, resective or disconnective surgery was performed to control ES. Frontal, frontotemporal, or parietal lobe surgery was performed in six cases based on predominant epileptic activities in those lobes. Posterior quadrantectomy^18^ was performed in two cases based on predominant epileptic activities in the posterior cortex, and hemispherotomy was performed in five cases who developed diffuse hemispheric epileptic activities with contralateral hemiparesis. Multilobar disconnective surgery preserving the central cortex (subtotal hemispherotomy)^5^ was performed in three cases who developed diffuse hemispheric epileptic activities without hemiparesis. An interval from CC to subsequent resective surgery ranged from 5 to 91 months (median 14.5 months). No unexpected postoperative complications occurred except those predicted before surgery such as hemiparesis and hemianopia in cases with multilobar disconnective surgeries and hemispherotomy. After surgery, all the patients accomplished freedom from ES through antiseizure medicines, indicating that the responsible hemisphere for their residual ES after CC was precisely identified.

### 2.2 Description of ES before and after CC

Video‐EEG monitoring was performed repeatedly for at least 12 hours in each patient. This study analyzed video‐EEG data collected just before CC and resective surgery. The EEG was recorded using NicVue (Natus Medical Incorporated) with electrodes placed according to the international 10–20 system. In some patients, electromyography (EMG) was simultaneously referred from the bilateral deltoid muscle.

To investigate the correlation between clinical expression of ES and the responsible hemisphere, we visually reviewed the recorded video‐EEG. Whereas we analyzed electroclinical ES with ictal behaviors on video‐EEG or EMG. An independent reviewer (DU), who was blinded to the patients' treatment, documented the behavioral ES without any clinical information about those patients. If the behaviors could not be evaluated due to poor recording resolution or patient's posture, eg lateral position on the bed, those were scored as “unknown” and excluded from the analysis. When ictal movements were subtle and laterality was difficult to assess, senior physicians (TO, RH, and YW) evaluated the characteristic of the ES and a consensus decision of all three senior physicians was taken into consideration. If even one of the three did not agree, those ES were excluded from the analysis. In particular, NF, MCU, and MCL were described by focusing on their laterality, ie “symmetric” or “asymmetric,” and “left” or “right.” We counted the number of NF, MCU, and MCL, but all those expressions were not always accompanied simultaneously in individual ES. For example, some seizures comprised only NF, while others could only display MCU and/or MCL. Therefore, the number of individual ictal movements is not the same even in the same patient.

**FIGURE 1:**
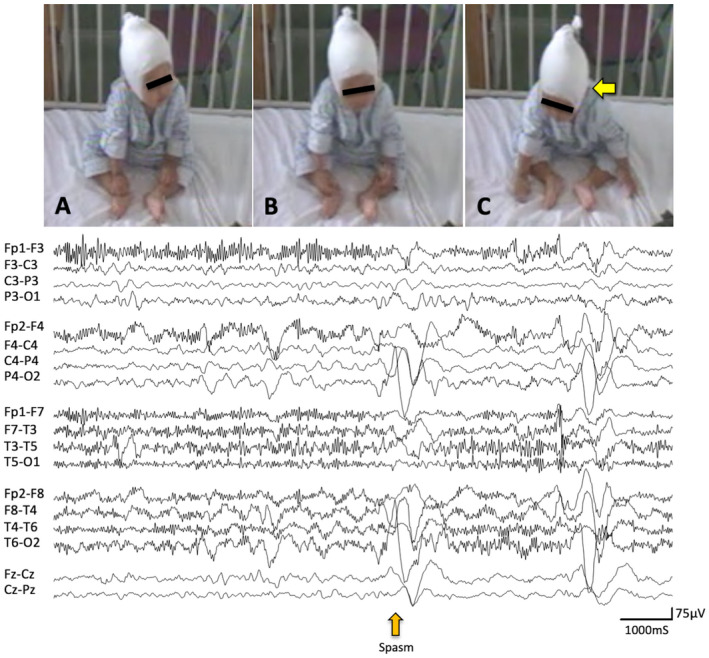
Asymmetric NF and MCU after CC (Case 4). Representative asymmetric NF and MCU are shown (case 4). Ictal correlates with a high‐amplitude slow wave in the right hemisphere followed by a brief electrodecremental pattern as seen on EEG (bottom). (A) Before the appearance of ES. (B,C) NF with slight head flexion and right side turning (arrow), where the body also flexed ventrally, and the left arm slightly moved laterally by the flexion of the shoulder joint (Video S1). NF, neck flexion; MCU, muscle contraction of upper extremities; CC, corpus callosotomy; EEG, electroencephalography; ES, epileptic spasms

**FIGURE 2:**
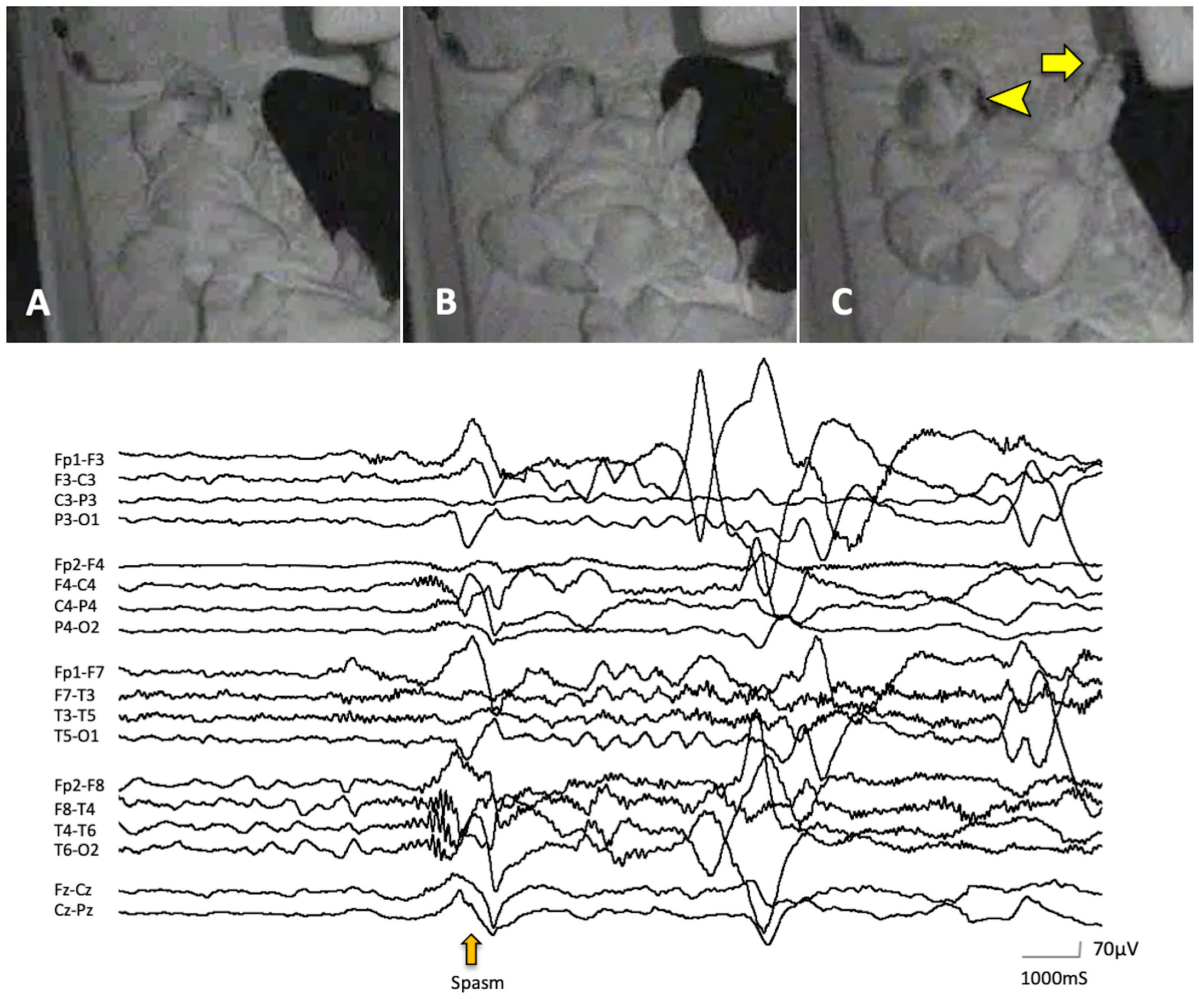
Asymmetric NF and MCU after CC (Case 12). Representative asymmetric NF and MCU are shown (case 12). Ictal correlates with fast waves and high‐amplitude slow wave in right hemisphere followed by brief electrodecremental pattern are seen on EEG (bottom). (A) Before the appearance of ES. (B,C) NF with forced head flexion and right side turning (arrowhead), where the body also flexed ventrally, and the left arm moved laterally by the flexion of the shoulder and elbow joints (arrow) (Video S2). NF, neck flexion; MCU, muscle contraction of upper extremities; CC, corpus callosotomy; EEG, electroencephalography; ES, epileptic spasms.

Asymmetric NF was characterized by neck flexion with the head turning or falling to one side (Figures 1 and 2). Asymmetric MCU and MCL were characterized by the unilaterally predominant muscular contractions involving the shoulder, arm, and leg (Figures 1 and 2). Supplemental video data are also available online for those ictal movements (Video S1 and S2). The spasms characteristics vary depending on the muscle groups' involvement and body postures.^2^ In fact, individual or group muscular contractions could not always be documented in detail. Usually, when analyzed by video, spasms captured in the front sitting or supine position were the easiest to score the symmetry/asymmetry of the movements. In the upper extremity, the abduction or flexion (forward or lateral movement) of the shoulder joint and flexion of the elbow joint were most frequently seen. In the lower extremity, the hip and knee joints were usually flexed. Thus, most of the analyzed spasms were flexor spasms. However, we observed some spasms accompanied movements in which the shoulder joints were flexed while the elbow joints were kept extended. In such cases, it might be flexor + extensor spasms, but it is impossible to make a clear classification.

ES was also confirmed by correlates of EEG including high voltage positive slow‐wave, fast activity, and/or decremental background attenuation^19^, ^20^ (Figures 1 and 2). However, the laterality of those ictal correlates was not always documented in this study. We counted the number of all electroclinical ES in each patient. However, subtle ES including motionless staring^20^ and rapid eye movements were excluded from the observation in this study since those are difficult to be described precisely due to the limited resolution of our video data. In some patients, ES appeared repetitively and developed to larger movements later in an event. We defined them as a cluster if each of them was comprised not less than five ES in this study. We observed up to twenty consecutive ES from the onset of visible movement for each cluster and included the first ten ES for statistical analysis to reduce the disparity of total counts of ES per patient.

All statistical analyses were performed with EZR (Saitama Medical Center, Jichi Medical University),^21^ a graphical user interface of R (The R Foundation for Statistical Computing). The chi‐square test or Fisher's exact test was used for statistical analysis. Statistical significance was defined at *P* < 0.001. In the contingency table, basic statistics are shown, including odds ratios and 95% confidence intervals (CI).

### 2.3 Scoring symmetric and asymmetric expressions

Neck flexion, MCU, and MCL in all ES were scored in each patient focusing on their symmetry/asymmetry and laterality. The direction of NF was defined as the direction to which the neck flexed or the head turned during ES. If the direction of NF deviated toward the left or right side, NF was scored as asymmetrically right or left, respectively (ASR and ASL, respectively). If it was straight to the middle, NF was scored as symmetric (S). For MCU and MCL, the predominant side was defined as the side in which the unilateral extremity was involved (the shoulder and arm for MCU and the leg for MCL). If the predominant side was right or left, MCU and MCL were scored as asymmetrically right or left, respectively (ASR and ASL, respectively). Even when the upper or lower extremities moved bilaterally, if the momentum on one side was larger than the other, we scored it as asymmetric. MCU and MCL were scored as symmetric (S) if they were symmetrically involved.

Then, the number of symmetric movements (N_S_) and asymmetrically right‐ and left‐predominant movements (N_ASR_ and N_ASL_, respectively) were counted separately before and after surgery. Finally, the relative frequency of symmetric movements and laterality index of asymmetric movements were calculated as follows,

$\left(\text{Relative frequency of symmetric movements}\right)=\frac{\left(N_{S}\right)}{\left(N_{S}+N_{\mathrm{ASL}}+N_{\mathrm{ASR}}\right)}\;$

$\left(\text{Laterality index of asymmetric movements}\right)=\frac{\left(N_{\mathrm{ASR}}‐N_{\mathrm{ASL}}\right)}{\left(N_{S}+N_{\mathrm{ASL}}+N_{\mathrm{ASR}}\right)}\;$

**FIGURE 3:**
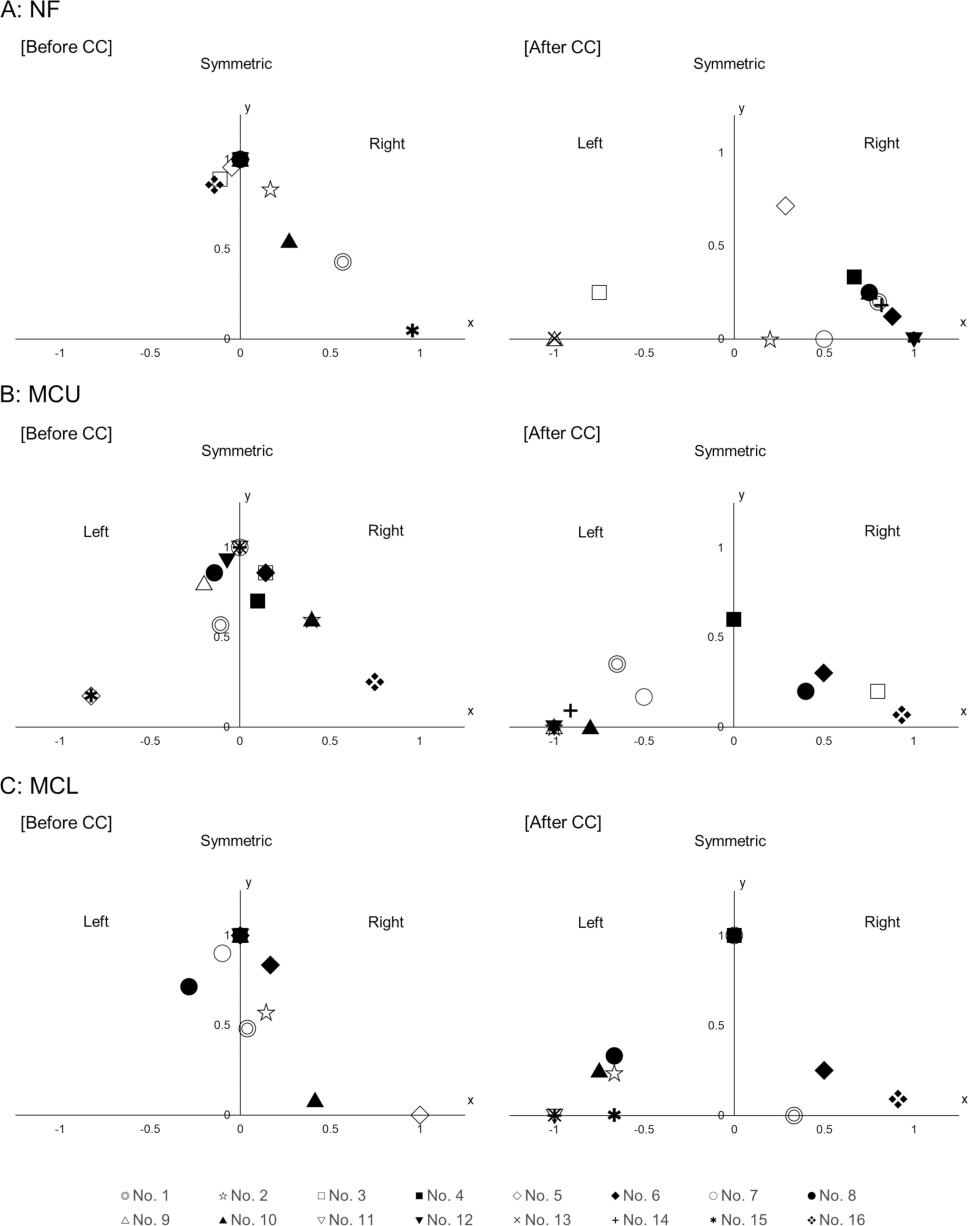
Symmetry and asymmetry in NF, MCU, and MCL before and after CC. X‐axis, relative laterality index of asymmetric movements; Y‐axis, the relative frequency of symmetric movements. Individual symbols correspond to each patient. The distribution of plots can be compared before and after the CC, and symmetry or laterality in each movement can be visualized here. Significant lateralization is defined if the absolute value of the laterality index is more than 0.5. Symbols show individual patients. (A) NF: Direction of NF during ES is straight to the middle before but deviated to the left or right side after CC in most patients, where significant lateralization to the responsible hemisphere is seen in nine of 12 patients. (B) MCU: MCU during ES is bilaterally symmetric but unilaterally predominant in most patients after CC, where significant lateralization contralateral to the responsible hemisphere is seen in 11 of 11 patients. (C) MCL: MCL during ES is bilaterally symmetric before but unilaterally predominant in many patients after CC, where significant lateralization contralateral to the responsible hemisphere is seen in seven of nine patients. NF, neck flexion; MCU, muscle contraction of upper extremities; MCL, muscle contraction of lower extremities; CC, corpus callosotomy; EEG, electroencephalography; ES, epileptic spasms.

To visually identify the changes in symmetry or asymmetry for each movement before and after CC, these calculated values were plotted on the graph as follows, (Figure 3A–C)

X = (Relative laterality index of asymmetric movements)

Y = (Relative frequency of symmetric movements)

Here, it means that all movements were seen on the right side if (X, Y) = (1, 0) and on the left side if (X, Y) = (−1, 0). Further, if (X, Y) = (0, 1), it means that all movements are symmetrical (Figure 3). It was defined as significant lateralization if the absolute value of the laterality index is above 0.5.

## 3 RESULTS

Table 2 summarizes the number and percentage of ES with each symmetric or asymmetric movement before and after CC. Table S1 shows the number of each ictal movement by the patient. Additionally, the individual relative frequency of symmetric movements and the laterality index of asymmetric movements are shown in Figure 3 (A, NF; B, MCU; C, MCL).

**TABLE 2:** The number and rate of the symmetric and asymmetric ES before and after CC

	Before CC	After CC	Odds ratio (95% CI) *P*‐value
Overall (n = 16)			
NF			
Symmetric	167 spasms (79.9%, 14 patients)	22 spasms (17.1%, 8 patients)	19.3 (10.9–34.2) *P* < 0.001
Asymmetric	42 spasms (20.1%, 7 patients)	107 spasms (82.9%, 14 patients)	
MCU			
Symmetric	145 spasms (60.7%, 16 patients)	29 spasms (19%, 8 patients)	6.6 (4.1–10.7) *P* < 0.001
Asymmetric	94 spasms (39.3%, 12 patients)	124 spasms (81%, 13 patients)	
MCL			
Symmetric	94 spasms (70.1%, 13 patients)	24 spasms (22.4%, 7 patients)	8.1 (4.5–14.6) *P* < 0.001
Asymmetric	40 spasms (29.9%, 7 patients)	83 spasms (77.6%, 10 patients)	
MRI^a^			
Unilateral MCD (n = 3)			
NF			
Symmetric	7 spasms (87.5%, 2 patients)	3 spasms (8.8%, 1 patient)	72.3 (6.5–803.1) *P* < 0.001
Asymmetric	1 spasm (12.5%, 1 patient)	31 spasms (91.2%, 3 patients)	
MCU			
Symmetric	21 spasms (87.5%, 3 patients)	10 spasms (19.6%, 2 patients)	28.7 (7.1–115.6) *P* < 0.001
Asymmetric	3 spasms (12.5%, 2 patients)	41 spasms (80.4%, 3 patients)	
MCL			
Symmetric	18 spasms (78.3%, 3 patients)	10 spasms (26.3%, 3 patients)	10.1 (3.0–34.3) *P* < 0.001
Asymmetric	5 spasms (21.7%, 3 patients)	28 spasms (73.7%, 2 patients)	
Unilateral nonspecific lesion^b^ (n = 6)			
NF			
Symmetric	54 spasms (66.7%, 5 patients)	5 spasms (10.2%, 2 patients)	17.6 (6.3–49.5) *P* < 0.001
Asymmetric	27 spasms (33.3%, 2 patients)	44 spasms (89.8%, 5 patients)	
MCU			
Symmetric	48 spasms (64%, 6 patients)	1 spasm (2.9%, 1 patient)	58.7 (7.6–453.3) *P* < 0.001
Asymmetric	27 spasms (36%, 3 patients)	33 spasms (97.1%, 4 patients)	
MCL			
Symmetric	19 spasms (63.3%, 4 patients)	3 spasms (12%, 2 patients)	12.7 (3.1–52.2) *P* < 0.001
Asymmetric	11 spasms (36.7%, 1 patient)	22 spasms (88%, 5 patients)	
No lesion (n = 6)			
NF			
Symmetric	102 spasms (87.9%, 6 patients)	13 spasms (31%, 4 patients)	16.3 (6.9–38.4) *P* < 0.001
Asymmetric	14 spasms (12.1%, 4 patients)	29 spasms (69%, 5 patients)	
MCU			
Symmetric	58 spasms (48.7%, 6 patients)	15 spasms (28.3%, 4 patients)	2.4 (1.2–4.8) *P* < 0.05
Asymmetric	61 spasms (51.3%, 6 patients)	38 spasms (71.7%, 5 patients)	
MCL			
Symmetric	32 spasms (69.6%, 5 patients)	1 spasm (7.1%, 1 patient)	29.7 (3.5–249.7) *P* < 0.001
Asymmetric	14 spasms (30.4%, 2 patients)	13 spasms (92.9%, 2 patients)	
Pre‐CC EEG epileptiform discharges			
With laterality (n = 8)			
NF			
Symmetric	60 spasms (62.5%, 7 patients)	11 spasms (14.3%, 5 patients)	10 (4.7–21.4) *P* < 0.001
Asymmetric	36 spasms (37.5%, 4 patients)	66 spasms (85.7%, 8 patients)	
MCU			
Symmetric	87 spasms (64.9%, 8 patients)	12 spasms (16.2%, 4 patients)	9.6 (4.7–19.5) *P* < 0.001
Asymmetric	47 spasms (35.1%, 6 patients)	62 spasms (83.8%, 7 patients)	
MCL			
Symmetric	57 spasms (62.6%, 6 patients)	13 spasms (27.1%, 3 patients)	4.5 (2.1–9.7) *P* < 0.001
Asymmetric	34 spasms (37.4%, 3 patients)	35 spasms (72.9%, 6 patients)	
Without laterality (n = 8)			
NF			
Symmetric	107 spasms (94.7%, 7 patients)	11 spasms (21.2%, 3 patients)	66.5 (23.1–191.4) *P* < 0.001
Asymmetric	6 spasms (5.3%, 3 patients)	41 spasms (78.8%, 6 patients)	
MCU			
Symmetric	58 spasms (55.2%, 8 patients)	17 spasms (21.5%, 4 patients)	4.5 (2.3–8.7) *P* < 0.001
Asymmetric	47 spasms (44.8%, 6 patients)	62 spasms (78.5%, 6 patients)	
MCL			
Symmetric	37 spasms (86%, 7 patients)	11 spasms (18.6%, 4 patients)	26.9 (9.1–79.5) *P* < 0.001
Asymmetric	6 spasms (14%, 4 patients)	48 spasms (81.4%, 4 patients)	

Abbreviations: CC, corpus callosotomy; CI, confidence interval; NF, neck flexion; MCU, muscular contraction of the upper extremities; MCL, muscular contraction of the lower extremities; MCD, malformation of cortical development; EEG, electroencephalography.

^a^ One patient with bilateral nonspecific lesions is not included here (case 8).

^b^ Nonspecific lesions include mild lobar or hemispheric volume loss and subtle signal changes in the white matter.

### 3.1 Neck flexion (NF)

In terms of the overall number of ES, the percentage of asymmetric NF was significantly higher after CC than before CC (82.9% vs. 20.1%, respectively; odds ratio, 19.3; 95% CI, 10.9–34.2; *P* < 0.001; Table 2).

The relative frequency of symmetric NF decreased and the absolute value of the laterality index of asymmetric NF increased significantly after CC in 12 patients (Table 3, Figure 3A). The direction of asymmetric NF was ipsilateral to the side of the responsible hemisphere that was the resected side at the subsequent surgery with significant lateralization in 9 of 12 patients (75%, Table 3).

**TABLE 3:** The number and rate of the patients with significant asymmetric ES before and after CC

	Before CC	After CC	Odds ratio (95% CI) *P*‐value	Relationship between predominant movement^a^ and responsible hemisphere	
				Ipsilateral	Contralateral
Overall (n = 16)					
NF	2/14 patients (14.3%)	12/14 patients (85.7%)	36 (4.3‐299) *P* < 0.001	9/12 patients (75%)	3/12 patients (25%)
MCU	3/16 patients (18.8%)	11/13 patients (84.6%)	23.8 (3.4‐169.4) *P* < 0.001	0/11 patients (0%)	11/11 patients (100%)
MCL	1/14 patients (7.1%)	9/12 patients (75%)	39 (3.5‐437.5) *P* < 0.001	2/9 patients (22.2%)	7/9 patients (77.8%)
MRI lesion^b^					
Unilateral MCD (n = 3)					
NF	0/2 patients (0%)	2/3 patients (66.7%)		1/2 patients (50%)	1/2 patients (50%)
MCU	0/3 patients (0%)	3/3 patients (100%)		0/3 patients (0%)	3/3 patients (100%)
MCL	0/3 patients (0%)	2/3 patients (66.7%)		0/2 patients (0%)	2/2 patients (100%)
Unilateral nonspecific lesion^c^ (n = 6)					
NF	1/5 patients (20%)	5/5 patients (100%)		5/5 patients (100%)	0/5 patients (0%)
MCU	1/6 patients (16.7%)	4/4 patients (100%)		0/4 patients (0%)	4/4 patients (100%)
MCL	0/4 patients (0%)	5/6 patients (83.3%)		1/5 patients (20%)	4/5 patients (80%)
Nonlesion (n = 6)					
NF	1/6 patients (16.7%)	4/5 patients (80%)		3/4 patients (75%)	1/4 patients (25%)
MCU	2/6 patients (33.3%)	4/5 patients (80%)		0/4 patients (0%)	4/4 patients (100%)
MCL	1/6 patients (16.7%)	1/2 patients (50%)		0/1 patients (0%)	1/1 patients (100%)
Pre‐CC EEG epileptiform discharges					
With laterality (n = 8)					
NF	2/7 patients (28.6%)	8/8 patients (100%)		7/8 patients (87.5%)	1/8 patients (12.5%)
MCU	1/8 patients (12.5%)	6/7 patients (85.7%)		0/6 patients (0%)	6/6 patients (100%)
MCL	0/6 patients (0%)	5/7 patients (71.4%)		2/5 patients (40%)	3/5 patients (60%)
Without laterality (n = 8)					
NF	0/7 patients (0%)	4/6 patients (66.7%)		2/4 patients (50%)	2/4 patients (50%)
MCU	2/8 patients (25%)	5/6 patients (83.3%)		0/5 patients (0%)	5/5 patients (100%)
MCL	1/8 patients (12.5%)	4/5 patients (80%)		0/4 patients (0%)	4/4 patients (100%)

Abbreviations: CC, corpus callosotomy; CI, confidence interval; NF, neck flexion; MCU, muscular contraction of the upper extremities; MCL, muscular contraction of the lower extremities. MCD, malformation of cortical development; EEG, electroencephalography.

^a^ Predominant movement includes the direction of NF and the predominant side of MCU and MCL.

^b^ One patient with bilateral nonspecific lesions is not included here (case 8).

^c^ Nonspecific lesions include mild lobar or hemispheric volume loss and subtle signal changes in the matter.

### 3.2 Muscular contraction of the upper extremity (MCU)

In terms of the total number of ES, the percentage of asymmetric MCU was significantly higher after CC than before CC (81% vs. 39.3% respectively; odds ratio 6.6; 95% CI 4.1–10.7; *P* < 0.001; Table 2).

The relative frequency of symmetric MCU decreased and the absolute value of the laterality index of asymmetric MCU increased significantly after CC in 11 patients (Table 3, Figure 3B). The predominant side of MCU was contralateral to the side of the responsible hemisphere with significant lateralization in all 11 patients (100%, Table 3).

### 3.3 Muscular contraction of the lower extremities (MCL)

In terms of the total number of ES, the percentage of asymmetric MCL was significantly higher after CC than before CC (77.6% vs. 29.9%, respectively; odds ratio 8.1; 95% CI 4.5–14.6; *P* < 0.001; Table 2).

The relative frequency of symmetric MCL decreased and the absolute value of the laterality index of asymmetric MCL increased significantly after CC in nine patients (Table 3, Figure 3C). The predominant side of MCL was contralateral to the side of the responsible hemisphere with significant lateralization in seven patients (77.8%, Table 3).

### 3.4 ES occurred in a cluster

Asymmetric NF was seen during a cluster in four patients before CC (data not shown) and seven patients after CC (Table S2). The direction of NF was consistently ipsilateral to the side of the responsible hemisphere throughout a cluster only in two patients (cases 13 and 15). In other patients, symmetry of NF gradually disappeared or developed in each cluster (cases 1, 4, 5, 6, and 14). However, initial or developed laterality during a cluster was consistent with the responsible hemisphere in four patients.

Asymmetric MCU was seen during a cluster in seven patients (data not shown) before CC and in nine patients after CC (Table S2). The predominant side of MCU was consistently contralateral to the side of the responsible hemisphere throughout a cluster in five patients (cases 2, 10, and 14–16). The MCU symmetry gradually disappeared or developed in other four patients (cases 1,4, 6, and 8) and the predominant side of MCU was indicative of the responsible hemisphere in one patient in whom the laterality did not switch during a cluster.

Asymmetric MCL was identified during a cluster in four patients (data not shown) before CC and in six patients after CC (Table S2). Once asymmetric MCL appeared, the predominant side was consistently contralateral to the responsible hemisphere in only one patient. Asymmetric features often disappeared or developed in the other five patients (cases 2, 6, 8, 15, and 16), and the predominant side of MCL was indicative of the responsible hemisphere in three patients in whom the laterality did not reverse during a cluster.

### 3.5 Laterality on MRI and pre‐CC EEG

Preoperative MRI showed unilateral malformation of cortical development (MCD) in three patients (cases 2, 6, and 7; Table 1). Most ictal movements were bilaterally symmetric before CC (87.5% for NF and MCU and 78.3% for MCL; Table 2). However, after CC majority of those showed asymmetric expression (91.2% for NF, 80.4% for MCU, and 73.7% for MCL; Table 2). The responsible hemisphere for post‐CC ES was consistent with the side of the lesion in two of the three patients (cases 2 and 7).

Six patients also had a unilateral nonspecific lesion in single or multiple lobes (cases 10–15; Table 1). EEG discharges were also bilateral in all patients but unilaterally predominant in five (cases 10 and 12–15; Table 1). However, its laterality was not always consistent with the side of the MRI nonspecific lesion. Many pre‐CC ictal movements were symmetric (66.7% for NF, 64% for MCU, and 63.3% for MCL; Table 2). But after CC, a higher rate of asymmetric ictal movements (89.8% for NF, 97.1% for MCU, and 88% for MCL; Table 2) were observed. The responsible hemisphere for post‐CC ES was consistent with the side of the lesion in all patients. However, the predominant side of pre‐CC epileptiform discharges was paradoxically contralateral to the side of the lesion in two of five patients (cases 14 and 15; Table 1).

There were eight patients with and without laterality in epileptiform discharges on pre‐CC EEG (Table 1). Even if there was laterality in pre‐CC epileptiform discharges, many ictal movements were still symmetric (62.5% for NF, 64.9% for MCU, and 62.6% for MCL; Table 2). Again, most post‐CC ictal movements showed asymmetric expressions (85.7% for NF, 83.8% for MCU, and 72.9% for MCL; Table 2). The responsible hemisphere for post‐CC ES was not consistent with the predominant side of pre‐CC epileptiform discharges in four of eight patients (cases 1, 3, 14, and 15; Table 1).

Preoperative functional neuroimaging was complicated to be interpreted and was not described in detail here due to differences in the modality (SPECT or PET), the distribution (focal or diffuse), and the patterns (hyper‐ or hypo‐accumulation). However, as with MRI and EEG, the pre‐CC laterality in their findings was not associated with the asymmetry of pre‐CC ictal movements that was finally clarified after CC (data not shown). Of seven patients who showed the laterality on functional neuroimaging before CC, the side of hypo‐accumulation on SPECT or PET was consistent with the responsible side of post‐CC ictal movements in five cases (cases 1, 2, 9, 13, and 15; Table 1).

## 4 DISCUSSION

We confirmed that residual ES after CC represents more asymmetric expression than before CC in selected patients who achieved seizure freedom by subsequent resective surgery. Among those patients, asymmetric NF, MCU, and MCL were suggested to be lateralizing signs for the responsible hemisphere. Our findings indicate that the residual epileptic region or lobe can be found in the hemisphere ipsilateral to the direction of NF and contralateral to the predominant side of MCU and MCL.

CC is an alternative option in the cases with diffuse epileptic discharges, suggesting contraindication for resective surgery, but the rate of seizure freedom was reported 10%–40%.^7^, ^8^, ^10^ Therefore, many patients have residual seizures even after the procedure. However, CC sometimes reveals epileptic foci, which were unidentified before surgery.^12^ This enables subsequent resective surgery providing better seizure outcomes in selected patients with residual seizures, including ES.^9^, ^12^, ^13^, ^14^

Criteria for subsequent resective surgery after CC has not been fully elucidated but are identical to those of conventional resective surgery, ie, focality or lateralization on EEG and neuroimages such as MRI, PET and SPECT, and asymmetric seizure semiology suggesting a focal onset.^12^ Although there has been a few studies concerning post‐CC seizure semiology, ES and head drop, and tonic posturing, have been documented to be asymmetric after CC.^12^ In this study, we focused on the asymmetry of ictal movements involved in ES after CC. All patients included in the study achieved seizure freedom after subsequent resective surgery, which could be linked to the seizure focus or the affected hemisphere and asymmetric ES after CC.^22^

Considering the brain's functional anatomy, it is reasonable that unilateral MCU and MCL persist after CC if the contralateral hemisphere is still affected. Some studies also reported that asymmetric muscular contraction of extremities is associated with contralateral ictal EEG discharges even in ES.^23^, ^24^

However, the precise mechanism of asymmetric NF clarified after CC is still unknown. The muscles complex including sternocleidomastoid, trapezius, and deltoid muscles are usually involved during ES.^23^, ^25^, ^26^ The sternocleidomastoid muscle is the functionally important muscle for NF. The contraction of unilateral sternocleidomastoid muscle induces the head toward the contralateral side.^27^, ^28^, ^29^ Corticomotor projection to the sternocleidomastoid muscle may be bilateral but predominantly contralateral according to the studies using magnetic transcranial stimulation.^30^, ^31^ These results may be accountable for the ipsilateral direction of NF to residual epileptic foci or hemisphere after CC.

The pathophysiology of ES and its related disorders is still controversial, but the cerebral cortex and subcortical structures including the brainstem have been suggested to play an important role in the generation of seizures and epileptic activities cooperatively or exclusively.^23^, ^24^, ^32^, ^33^, ^34^, ^35^ In terms of background EEG abnormality in West syndrome, Pinard et al and Baba et al reported bilateral hypsarrhythmia frequently seen in the disorder altered into unilateral after CC.^10^, ^36^ Baba et al also quantitatively studied the EEG in hypsarrhythmia before and after CC in patients with West syndrome and concluded that the surgical outcome depended on the power of fast oscillations and its connectivity with slow waves suggesting an epileptic abnormality in the cerebral cortex and the subcortical structures.^15^ Residual epileptic discharges in one hemisphere after CC mean that cortical epileptogenicity exists primarily in the unilateral hemisphere, and the corpus callosum is involved as an interhemispheric modulator preoperatively.^15^, ^37^ In patients in this study, not only interictal EEG discharges but also ictal expressions in ES turned into more asymmetric ones after CC, and ES disappeared after subsequent unilateral resective surgery. This may imply the larger contribution of the cerebral cortex and the corpus callosum in the generation of bilaterally expressed ES before CC.

Few studies have reported the feature of clusters of ES. It has been known that the intensity of movements wax and wane during a cluster of ES.^2^ It is interesting that the asymmetric movements gradually turned into symmetric ones after the initiation or in the end part of a cluster of ES in some cases after CC. This was more likely to occur with NF than with MCU and MCL. The pathophysiology of this finding has been unclear, but our results suggest that intracortical recruitment of seizure activity may be most important for the cluster of ES. Kobayashi et al. observed that the leading unilateral spikes disappeared in the middle to end parts of the bursts of bilateral spike‐wave discharges in secondary bilateral synchrony.^38^ Thus, similar rationales such as cortico‐subcortical‐cortical networks may also be involved in the generation of symmetric ES at different stages of a cluster. Contrarily, the distribution of the ipsilateral corticospinal tracts, which accounts for 10% of the entire corticospinal tracts and ipsilaterally terminates to the muscles of the trunk, neck, and shoulders, also may be involved in this phenomenon. Kobayashi et al. showed that even unilateral ictal correlates (slow waves and gamma activity) on EEG could trigger both unilateral and bilateral deltoid muscle contraction in selected patients with ES.^39^ Although the predominant side of MCU and MCL were switched right and left in a few patients even after CC, the mechanism for this is still unclear.

In this study, we also attempted to correlate the symmetry/asymmetry of pre‐CC ES with preoperative objective findings such as specific or nonspecific lesions on MRI and laterality of epileptiform discharges on EEG suggestive of focal onset ES. However, all ictal movements of ES were primarily symmetric before CC, and asymmetric expressions were clarified only after CC, irrespective of pre‐CC objective findings.

## 5 LIMITATIONS

There are some limitations to this study. First, this study did not include the patients who did not undergo CC and those with bilateral epileptic focus even post‐CC who can display bilaterally independent asymmetric ES. In this study, resected or disconnected cortical region/hemisphere is exclusively responsible for post‐CC ES because all patients achieved freedom from ES by subsequent resective surgery. This is an advantage of the study. However, it is still unknown whether all patients, particularly those with bilaterally diffuse epileptic focus, show asymmetric features after CC.

Second, electromyography placed on the deltoid muscles was recorded in some patients, but the findings were only used to assist visual analyses of video data in this study as it was not routinely done in earlier cases at our institution. To confirm the variability of muscular participation, electromyography from the multiple muscles may be useful.^40^

Third, the laterality of ictal EEG was not correlated to each movement in ES. Some investigative studies have revealed laterality of ictal correlates, including time‐frequency analysis of ictal fast activity and measurement of slow‐wave component time‐locked with electromyography.^19^, ^39^ However, it is usually difficult to find electrographic focus on conventional scalp EEG in ES.

Finally, the number of patients included in this study is small. Furthermore, this is a retrospective study; therefore, the sensitivity and specificity of the lateralizing signs were undetected. Although the results in this study are informative, further study should be planned to correlate other factors including EEG and EMG, and test a hypothesis and usefulness as a lateralizing sign. As this is a single‐center study, only few patients were candidates for this type of surgery, thus long‐term prospective multicenter study will be needed in the future.

## 6 CONCLUSION

Asymmetric NF, MCU, and MCL were less frequent before but became remarkable and frequent after CC in patients with ES. The direction of NF was ipsilateral, and the predominant side of MCU and MCL was contralateral to the responsible hemisphere. More attention to asymmetric features of ES should be paid to patients who underwent CC in order not to lose a chance of seizure freedom by the subsequent resective surgery.

This study suggests that the cerebral cortex generates symmetric and asymmetric ES even if it is unilaterally affected, and the corpus callosum is suggested to be involved in the symmetric expression of ES in some patients with West syndrome.

## CONFLICT OF INTEREST

None of the authors has any conflict of interest to disclose. Dr. Tomonori Ono is on the editorial board of Epilepsia Open.

## ETHICAL PUBLICATION STATEMENT

We confirm that we have read the Journal's position on issues involved in ethical publication and affirm that this report is consistent with those guidelines.

## Supporting information


Table S1

Table S2

Video S1

Video S2
