# Supplementary material for: Asymmetric epileptic spasms after corpus callosotomy in children with West syndrome may be a good indicator for unilateral epileptic focus and subsequent resective surgery
Source: Epilepsia Open. 2022 Aug 1;7(3):474–87. doi: 10.1002/epi4.12631 (PMC9436295; doi:10.1002/epi4.12631)
Supplement: Supplementary file 1 — Table S1 [file EPI4-7-474-s003.docx]

| Patient | Before CC | | | | | After CC | | | | |
| --- | --- | --- | --- | --- | --- | --- | --- | --- | --- | --- |
| NF | Cluster | S | ASR | ASL | Laterality index* | Cluster | S | ASR | ASL | Laterality index* |
| 1 | + | 6 | 8 | 0 | 0.57 | + | 4 | 16 | 0 | 0.8 |
| 2 |  | 5 | 1 | 0 | 0.17 |  | 0 | 3 | 2 | 0.2 |
| 3 |  | 8 | 0 | 1 | 0.11 |  | 1 | 0 | 3 | 0.75 |
| 4 | + | 13 | 0 | 0 | 0 | + | 3 | 6 | 0 | 0.67 |
| 5 | + | 62 | 0 | 3 | 0.05 | + | 5 | 2 | 0 | 0.29 |
| 6 |  | 2 | 0 | 0 | 0 | + | 3 | 22 | 0 | 0.88 |
| 7 |  | 0 | 0 | 0 | NA |  | 0 | 3 | 1 | 0.5 |
| 8 |  | 4 | 0 | 0 | 0 |  | 1 | 3 | 0 | 0.75 |
| 9 |  | 1 | 0 | 0 | 0 |  | 0 | 0 | 2 | 1 |
| 10 | + | 6 | 4 | 1 | 0.27 |  | 1 | 3 | 0 | 0.75 |
| 11 | + | 12 | 0 | 0 | 0 |  | 0 | 0 | 0 | NA |
| 12 |  | 0 | 0 | 0 | NA |  | 0 | 8 | 0 | 1 |
| 13 | + | 16 | 0 | 0 | 0 | + | 0 | 0 | 10 | 1 |
| 14 |  | 19 | 0 | 0 | 0 | + | 4 | 18 | 0 | 0.82 |
| 15 | + | 1 | 22 | 0 | 0.96 | + | 0 | 5 | 0 | 1 |
| 16 |  | 12 | 0 | 2 | 0.14 |  | 0 | 0 | 0 | NA |
| MCU |  | S | ASR | ASL | Laterality index* |  | S | ASR | ASL | Laterality index* |
| 1 | + | 21 | 6 | 10 | 0.11 | + | 7 | 0 | 13 | 0.65 |
| 2 |  | 3 | 2 | 0 | 0.4 | + | 0 | 0 | 15 | 1 |
| 3 |  | 6 | 1 | 0 | 0.14 |  | 1 | 4 | 0 | 0.8 |
| 4 | + | 14 | 4 | 2 | 0.1 | + | 6 | 2 | 2 | 0 |
| 5 | + | 5 | 0 | 24 | 0.83 |  | 0 | 0 | 0 | NA |
| 6 |  | 6 | 1 | 0 | 0.14 | + | 9 | 18 | 3 | 0.5 |
| 7 | + | 12 | 0 | 0 | 0 |  | 1 | 1 | 4 | 0.5 |
| 8 | + | 18 | 0 | 3 | 0.14 | + | 3 | 9 | 3 | 0.4 |
| 9 | + | 8 | 0 | 2 | 0.2 |  | 0 | 0 | 3 | 1 |
| 10 | + | 3 | 2 | 0 | 0.4 | + | 0 | 1 | 9 | 0.8 |
| 11 |  | 6 | 0 | 0 | 0 |  | 0 | 0 | 0 | NA |
| 12 | + | 13 | 0 | 1 | 0.07 |  | 0 | 0 | 8 | 1 |
| 13 |  | 2 | 0 | 0 | 0 |  | 0 | 0 | 0 | NA |
| 14 | + | 19 | 0 | 0 | 0 | + | 1 | 0 | 10 | 0.91 |
| 15 | + | 5 | 0 | 24 | 0.83 | + | 0 | 0 | 5 | 1 |
| 16 |  | 4 | 12 | 0 | 0.75 | + | 1 | 14 | 0 | 0.93 |
| MCL |  | S | ASR | ASL | Laterality index* |  | S | ASR | ASL | Laterality index* |
| 1 | + | 12 | 7 | 6 | 0.04 |  | 0 | 2 | 1 | 0.33 |
| 2 |  | 4 | 2 | 1 | 0.14 | + | 5 | 1 | 15 | 0.67 |
| 3 |  | 2 | 0 | 0 | 0 |  | 0 | 0 | 0 | NA |
| 4 |  | 4 | 0 | 0 | 0 |  | 0 | 0 | 0 | NA |
| 5 |  | 0 | 1 | 0 | 1 |  | 0 | 0 | 0 | NA |
| 6 |  | 5 | 1 | 0 | 0.17 | + | 4 | 10 | 2 | 0.5 |
| 7 | + | 9 | 0 | 1 | 0.1 |  | 1 | 0 | 0 | 0 |
| 8 | + | 25 | 0 | 10 | 0.29 | + | 10 | 0 | 20 | 0.67 |
| 9 | + | 10 | 0 | 0 | 0 |  | 0 | 0 | 0 | NA |
| 10 | + | 1 | 8 | 3 | 0.42 |  | 1 | 0 | 3 | 0.75 |
| 11 |  | 1 | 0 | 0 | 0 | + | 0 | 0 | 10 | 1 |
| 12 | + | 13 | 0 | 0 | 0 |  | 2 | 0 | 0 | 0 |
| 13 |  | 0 | 0 | 0 | NA |  | 0 | 0 | 2 | 1 |
| 14 |  | 4 | 0 | 0 | 0 |  | 0 | 0 | 1 | 1 |
| 15 |  | 0 | 0 | 0 | NA | + | 0 | 1 | 5 | 0.67 |
| 16 |  | 4 | 0 | 0 | 0 | + | 1 | 10 | 0 | 0.91 |

**Table S1. The individual number of ES in which each symmetric/asymmetric ictal movement was observed**

ES, epileptic spasms; CC, corpus callosotomy; NF, neck flexion; MCU, muscular contraction of the upper extremities; MCL, muscular contraction of the lower extremities; Cluster (+), scored spasms include those in the cluster. S, symmetric; ASR, asymmetrically right; ASL, asymmetrically left; NA, not applicable. * The absolute value of laterality index is shown.
