# Supplementary material for: Asymmetric epileptic spasms after corpus callosotomy in children with West syndrome may be a good indicator for unilateral epileptic focus and subsequent resective surgery
Source: Epilepsia Open. 2022 Aug 1;7(3):474–87. doi: 10.1002/epi4.12631 (PMC9436295; doi:10.1002/epi4.12631)
Supplement: Supplementary file 2 — Table S2 [file EPI4-7-474-s001.docx]

| Patient-cluster # | 1 |  |  |  | 5 |  |  |  |  | 10 |  |  |  |  | 15 |  |  |  |  | 20 | Number of ES | Duration (s) | Responsible hemisphere |
| --- | --- | --- | --- | --- | --- | --- | --- | --- | --- | --- | --- | --- | --- | --- | --- | --- | --- | --- | --- | --- | --- | --- | --- |
| Direction of NF | | | | | | | | | | | | | | | | | | | | | | | |
| Case 1-1 | R | R | R | R | R | R | R | R | R | R | S | S | S | S | S | S |  |  |  |  | 16 | 167 | R |
| -2 | R | R | R | R | R | R | S | S | S | S | S | S | S | S | S | S | S | S | S | S | 20 | 197 |  |
| Case 4-1 | R | R | R | R | R | R | S | S | S |  |  |  |  |  |  |  |  |  |  |  | 9 | 63 | R |
| Case 5-1 | R | R | S | S | S | S | S |  |  |  |  |  |  |  |  |  |  |  |  |  | 7 | 140 | R |
| Case 6-1 | R | R | R | R | R | R |  |  |  |  |  |  |  |  |  |  |  |  |  |  | 6 | 47 | L |
| -2 | R | R | R | R | R |  |  |  |  |  |  |  |  |  |  |  |  |  |  |  | 5 | 34 |  |
| -3 | S | S | R | R | R | R |  |  |  |  |  |  |  |  |  |  |  |  |  |  | 6 | 93 |  |
| Case 13-1 | L | L | L | L | L | L | L | L | L | L |  |  |  |  |  |  |  |  |  |  | 10 | 130 | L |
| Case 14-1 | S | R | R | R | R | R | R | R | R | R | R |  |  |  |  |  |  |  |  |  | 11 | 223 | R |
| -2 | R | R | R | R | S | R | R |  |  |  |  |  |  |  |  |  |  |  |  |  | 7 | 83 |  |
| -3 | R | R | S | S | R |  |  |  |  |  |  |  |  |  |  |  |  |  |  |  | 5 | 103 |  |
| Case 15-1 | R | R | R | R | R |  |  |  |  |  |  |  |  |  |  |  |  |  |  |  | 5 | 211 | R |
| Predominant side of MCU | | | | | | | | | | | | | | | | | | | | | | | |
| Case 1-1 | S | L | L | L | L | L | L | L | L | L | L | L | L |  |  |  |  |  |  |  | 13 | 132 | R |
| -2 | L | S | S | L | L | L | S | S | S | S | S | L | S | S | S |  |  |  |  |  | 15 | 197 |  |
| Case 2-1 | L | L | L | L | L |  |  |  |  |  |  |  |  |  |  |  |  |  |  |  | 5 | 41 | R |
| -2 | L | L | L | L | L |  |  |  |  |  |  |  |  |  |  |  |  |  |  |  | 5 | 129 |  |
| Case 4-1 | R | R | S | S | S | S | L | S | L | S | R |  |  |  |  |  |  |  |  |  | 11 | 92 | R |
| Case 6-1 | S | L | R | S | R |  |  |  |  |  |  |  |  |  |  |  |  |  |  |  | 5 | 35 | L |
| -2 | S | S | S | S | R |  |  |  |  |  |  |  |  |  |  |  |  |  |  |  | 5 | 60 |  |
| -3 | R | S | R | R | L |  |  |  |  |  |  |  |  |  |  |  |  |  |  |  | 5 | 39 |  |
| -4 | R | R | R | R | L | S | R | R |  |  |  |  |  |  |  |  |  |  |  |  | 8 | 150 |  |
| Case 8-1 | L | L | S | S | S |  |  |  |  |  |  |  |  |  |  |  |  |  |  |  | 5 | 155 | L |
| -2 | R | R | R | R | R | R |  |  |  |  |  |  |  |  |  |  |  |  |  |  | 6 | 151 |  |
| Case 10-1 | L | L | L | L | L | L | L |  |  |  |  |  |  |  |  |  |  |  |  |  | 7 | 162 | R |
| Case 14-1 | L | L | L | L | L | L | L |  |  |  |  |  |  |  |  |  |  |  |  |  | 7 | 83 | R |
| Case 15-1 | L | L | L | L | L |  |  |  |  |  |  |  |  |  |  |  |  |  |  |  | 5 | 211 | R |
| Case 16-1 | R | R | R | R | R |  |  |  |  |  |  |  |  |  |  |  |  |  |  |  | 5 | 213 | L |
| -2 | R | R | R | R | R | R | R | R |  |  |  |  |  |  |  |  |  |  |  |  | 8 | 348 |  |
| Predominant side of MCL | | | | | | | | | | | | | | | | | | | | | | | |
| Case 2-1 | L | S | S | L | L |  |  |  |  |  |  |  |  |  |  |  |  |  |  |  | 5 | 117 | R |
| Case 6-1 | S | L | R | S | S |  |  |  |  |  |  |  |  |  |  |  |  |  |  |  | 5 | 35 | L |
| Case 8-1 | S | S | S | S | S | S | L | L | L |  |  |  |  |  |  |  |  |  |  |  | 9 | 123 | L |
| -2 | L | L | L | S | L | L | L |  |  |  |  |  |  |  |  |  |  |  |  |  | 7 | 367 |  |
| -3 | L | L | L | L | S | L | S | L | L | L |  |  |  |  |  |  |  |  |  |  | 10 | 214 |  |
| Case 11-1 | L | L | L | L | L | L | L | L | L | L | L | L | L | L | L | L | L | L | L | L | 20 | 137 | R |
| Case 15-1 | R | L | L | L | L | L |  |  |  |  |  |  |  |  |  |  |  |  |  |  | 6 | 211 | R |
| Case 16-1 | S | R | R | R | R | R | R |  |  |  |  |  |  |  |  |  |  |  |  |  | 7 | 348 | L |

**Table S2. Direction of NF and predominant side of MCU and MCL during each cluster of ES after CC**

NF, neck flexion; MCU, muscular contraction of the upper extremities; MCL, muscular contraction of the lower extremities; R, right; L, left; S, symmetric; ES, epileptic spasms; CC, corpus callosotomy.
